# Increased cognitive complexity reveals abnormal brain network activity in individuals with corpus callosum dysgenesis

**DOI:** 10.1016/j.nicl.2018.11.005

**Published:** 2018-11-14

**Authors:** Luke J. Hearne, Ryan J. Dean, Gail A. Robinson, Linda J. Richards, Jason B. Mattingley, Luca Cocchi

**Affiliations:** a Queensland Brain Institute, The University of Queensland, Brisbane, Queensland, Australia; b School of Psychology, The University of Queensland, Brisbane, Australia; c School of Biomedical Sciences, The University of Queensland, Brisbane, Australia; d Clincal Brain Networks Group, QIMR Berghofer Medical Research Institute, Brisbane, Queensland, Australia

**Keywords:** Connectivity, Brain networks, fMRI, Reasoning, Cognitive control, Connectome

## Abstract

Cognitive reasoning is thought to require functional interactions between whole-brain networks. Such networks rely on both cerebral hemispheres, with the corpus callosum providing cross-hemispheric communication. Here we used high-field functional magnetic resonance imaging (7 T fMRI), a well validated cognitive task, and brain network analyses to investigate the functional networks underlying cognitive reasoning in individuals with corpus callosum dysgenesis (CCD), an anatomical abnormality that affects the corpus callosum. Participants with CCD were asked to solve cognitive reasoning problems while their brain activity was measured using fMRI. The complexity of these problems was parametrically varied by changing the complexity of relations that needed to be established between shapes within each problem matrix. Behaviorally, participants showed a typical reduction in task performance as problem complexity increased. Task-evoked neural activity was observed in brain regions known to constitute two key cognitive control systems: the fronto-parietal and cingulo-opercular networks. Under low complexity demands, network topology and the patterns of local neural activity in the CCD group closely resembled those observed in neurotypical controls. By contrast, when asked to solve more complex problems, participants with CCD showed a reduction in neural activity and connectivity within the fronto-parietal network. These complexity-induced, as opposed to resting-state, differences in functional network activity help resolve the apparent paradox between preserved network architecture found at rest in CCD individuals, and the heterogeneous deficits they display in response to cognitive task demands [preprint: https://doi.org/10.1101/312629].

## Highlights

• Individuals with corpus callosum dysgenesis fail to develop a normal corpus callosum.
• Resting-state functional brain networks in callosal dysgenesis appear relatively normal.
• Cognitive complexity revealed a deficit in fronto-parietal network activity.
• Differences in brain activity might only be revealed when under cognitive load.

## 1 Introduction

The corpus callosum is the major white matter commissure of the brain. It connects the left and right cerebral hemispheres, and consists of more than 190 million axonal projections (Tomasch, 1954). These connections are thought to support the coupling of bilateral functional networks (Honey et al., 2009; Putnam et al., 2008; Roland et al., 2017; Shen et al., 2015; van der Knaap and van der Ham, 2011) that underpin both simple and complex behavior (Cocchi et al., 2014; Duncan, 2010; Schulte and Müller-Oehring, 2010). For example, adults who have had their corpus callosum surgically sectioned to control intractable seizures often experience a ‘disconnection syndrome’ in which they fail to complete tasks that require information-sharing between the two cerebral hemispheres (Gazzaniga et al., 1962; Seymour et al., 1994). By contrast, in individuals with *corpus callosum dysgenesis* (CCD), a neurodevelopmental condition in which the corpus callosum is only partially formed or where callosal axons fail to cross the cerebral midline (complete agenesis), deficits in behavioral measures of interhemispheric communication are generally more subtle (Lassonde et al., 1991; Paul et al., 2007).

The neuropsychological profile of those with CCD are heterogeneous, ranging from subtle cognitive deficits to severe impairments across a wide variety of cognitive domains (Paul et al., 2016; Paul et al., 2004; Romaniello et al., 2017; Siffredi et al., 2013). Investigations using functional magnetic resonance imaging (fMRI) have reported intact resting-state functional brain networks in individuals with CCD (e.g., the default-mode network) (Owen et al., 2013; Tyszka et al., 2011). Results from these studies suggest that the coupling of these networks is supported by alternative interhemispheric white matter pathways, such as those projecting through the anterior and posterior commissures (Lassonde et al., 1991; Tovar-Moll et al., 2014). However, such routes are indirect, polysynaptic and functionally costly (Marco et al., 2012; Owen et al., 2013).

The processing limits of functional networks shaped by CCD development are not well understood. Studies suggest that brain activity within sensory-motor networks during tactile stimulation and finger tapping tasks is preserved in CCD (Duquette et al., 2008; Quigley et al., 2003). However, less is known about the function of brain networks associated with higher cognitive abilities (e.g., language; Hinkley et al., 2016). In neurotypical individuals, the execution of complex cognitive tasks is associated with increased activity within, and coupling between, fronto-parietal (FPN) and cingulo-opercular networks (CON) (Cocchi et al., 2014; Crittenden et al., 2016; Duncan, 2010). Whether individuals with CCD produce similar task-evoked patterns of brain network activity, and to what degree indirect pathways can support functional network coupling as task demands increase, is not currently known. It is likely that normal brain network topology and activity will be detectable in states of low or absent cognitive load (e.g., in the resting-state) (Owen et al., 2013; Tovar-Moll et al., 2014; Tyszka et al., 2011). By contrast, altered topology and deficits in network activity may emerge as cognitive demands increase, revealing functional limits in brain network plasticity.

To test this hypothesis, we used high field (7 T) fMRI to characterize functional brain networks in seven individuals with CCD. We looked for changes in network configuration as individuals engaged in a well validated test of cognitive reasoning called the Latin Square Task (LST) (Birney et al., 2006). The LST is a non-verbal relational reasoning paradigm, similar to the popular game of Sudoku in its construction and rules, in which task complexity is parametrically varied with minimal demands on working memory (Birney et al., 2006; Halford and Phillips, 1998). We compared brain activation and network responses obtained in the seven CCD individuals with those from a normative sample of 30 neurologically typical participants. We identified specific functional connectivity deficits in CCD individuals, which only emerged under conditions of high cognitive demand.

## 2 Materials and methods

### 2.1 Participants

**Fig. 1:**
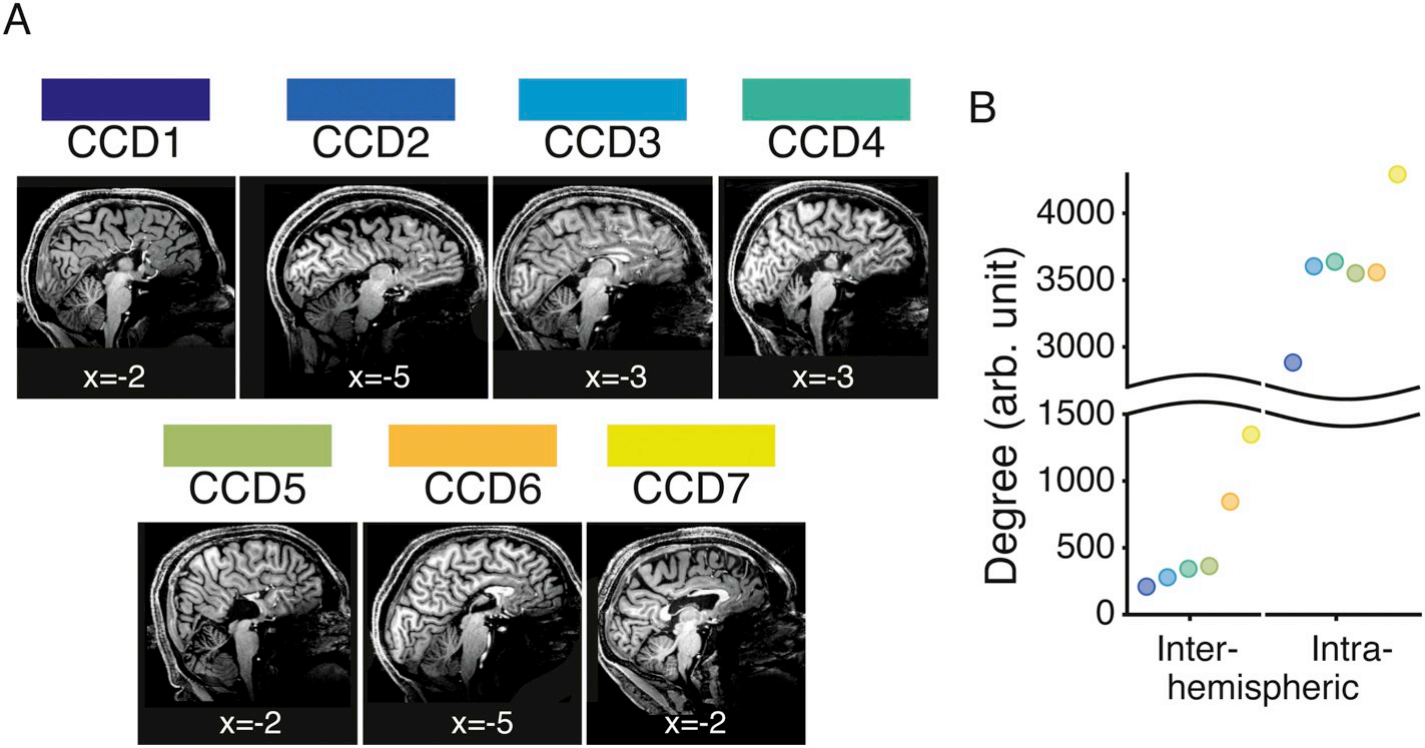
Structural anomalies in the seven corpus callosum dysgenesis (CCD) participants. A. Sagittal view (Montreal Neurological Institute, MNI, x coordinate indicated) of midline brain in individuals with CCD. Participants CCD1, CCD2 and CCD4 had complete agenesis of the corpus callosum. The other four participants had partial dysgenesis of the corpus callous with intact remnants: CCD3 had a preserved genu and posterior body; CCD5 had only a preserved body; CCD6 had an intact genu and rostrum remnant; and CCD7 had an intact genu, rostrum and splenium, as well as the anterior portion of the body. B. The number of structural connections across (inter) and within (intra) hemispheres was summed across ipsilateral and contralateral paired regions in each connectome (*N* = 6, CCD2 - CCD7). Dots are color-coded according to the convention depicted in panel A. As expected, participants with complete agenesis of the corpus callosum had fewer interhemispheric white matter connections (indicated by smaller numbers on the *y axis*) than those with a partially formed callosum. This trend was similar for intra-hemispheric connections. DWI data for CCD1 were not available due to a technical problem with data acquisition.

Participants were recruited prospectively between October 2014 to April 2016. Individuals with CCD enrolled in the Australian database of corpus callosum disorders. CCD participants resided in, or travelled to, the city of Brisbane to undertake cognitive assessments and high-field magnetic resonance imaging (7 T MRI) scans. The current study included seven participants with CCD aged 24–64 years (two females, one left handed participant, one ambidextrous) with levels of intelligence within the average range or above [Wechsler Abbreviated Scale of Intelligence, Full Scale IQ 88–124, mean = 102.29, SD = 13.35; see Table 1 for scores on additional measures of fluid (Advanced Raven's Progressive Matrices) and crystalized intelligence (National Adult Reading Test)]. Participants CCD1, CCD2 and CCD4 had complete agenesis of the corpus callosum, whereas the remaining individuals had partial agenesis (see Fig. 1A for a description of the corpus callosum abnormality). In line with previous work (Tovar-Moll et al., 2014), the CCD sample was heterogeneous, with different degrees of inter- and intra-hemispheric structural connectivity (Fig. 1B, see methods below).

**Table 1:** Demographics of the CCD participants and their scores on intellectual function measures.

	Age (Years)	Sex	VIQ (100 ± 15)	PIQ (100 ± 15)	FSIQ (100 ± 15)	NART IQ (100 ± 15)	APM (10.2 ± 1.4)	Corpus Callosum abnormality
CCD1	24	M	94	99	97	96	8	Complete
CCD2	40	M	88	103	96	96	5	Complete
CCD3	24	M	128	115	124	110	10	Partial: intact genu, posterior body remnant
CCD4	47	F	92	105	99	112	9	Complete
CCD5	46	M	91	88	88	113	7	Partial: body remnant
CCD6	64	M	108	127	118	112	11	Partial: intact genu and rostrum remnant
CCD7	24	F	91	80	94	100	5	Partial: intact genu, rostrum, splenium anterior body

Note: FSIQ, Full Scale Intelligence Quotient; VIQ, Verbal Intelligence Quotient; PIQ, Performance Intelligence Quotient; NART IQ, National Adult Reading Test derived premorbid Intelligence Quotient; APM, Raven's Advanced Progressive Matrices Set One. Typical mean and standard deviations are displayed underneath each test. For the APM these values were calculated from the 30 healthy individuals included in the study.

To benchmark participants' behavioral performance, their patterns of task-induced brain activity and their functional connectivity, we included a sample of 30 healthy controls (mean age = 23.60, SD = 3.95 years; 17 females, all right handed). The controls were a subsample of a previously published study (Hearne et al., 2017) who had completed the same task and imaging protocols. Individuals with the most comparable gender, age and behavioral performance profiles to the CCD individuals were included in the current cohort. These participants were not administered the neuropsychological battery (Table 1) but were compared in terms of their behavioral performance, fluid intelligence (Raven's Advanced Progressive Matrices), gender, handedness and age.

All methods and procedures were approved by The University of Queensland Human Research Ethics Committee and written informed consent was obtained from all participants.

### 2.2 Experimental design and statistical analysis

Participants completed three × 12-min runs of the Latin Square Task (LST) (Birney et al., 2006), high-angular resolution diffusion MRI, and an MP2RAGE anatomical scan.

#### 2.2.1 Task

**Fig. 2:**
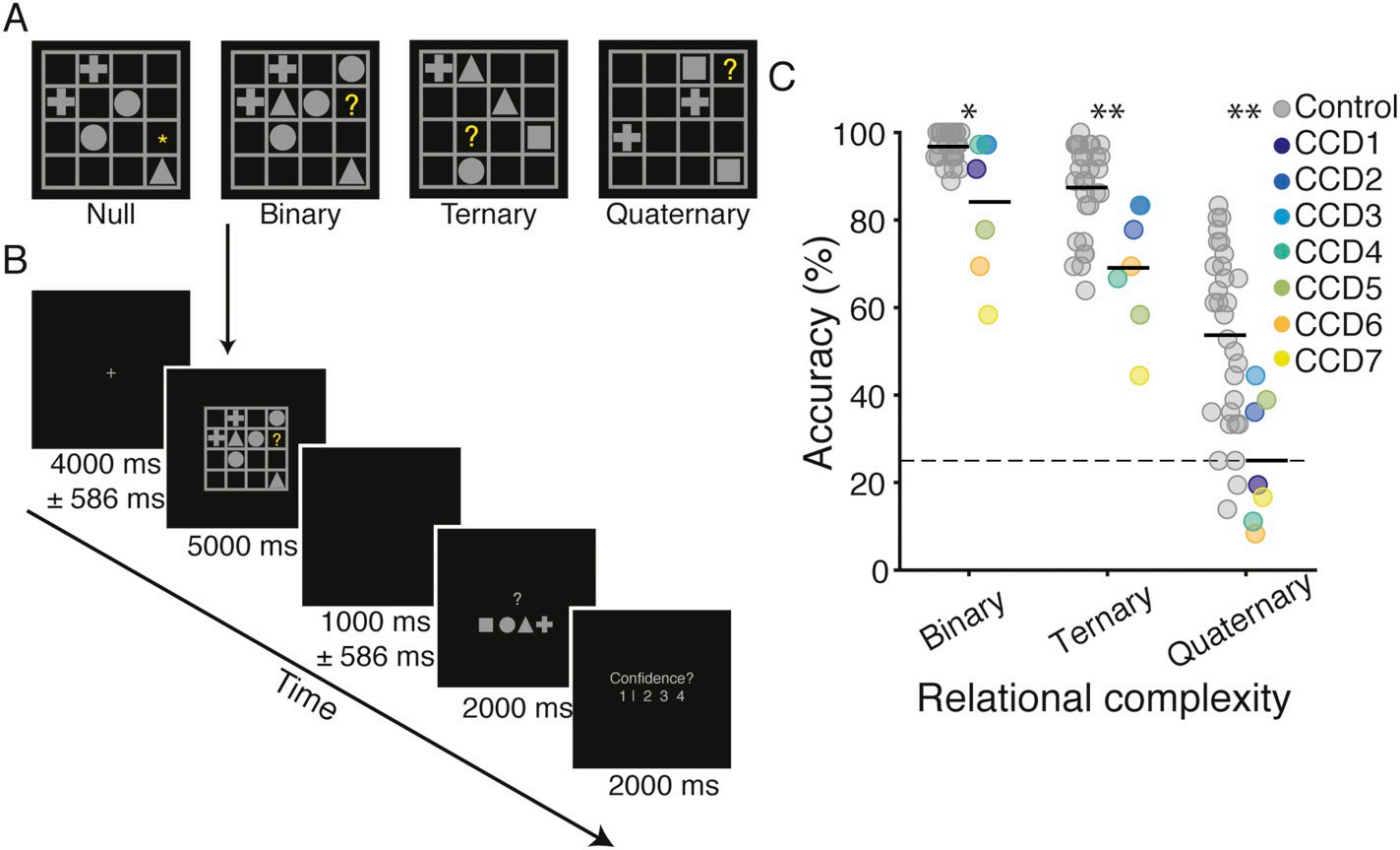
Latin Square Task design and behavioral performance of individuals with corpus callosum dysgenesis (N = 7) and controls (N = 30). A. Examples of each reasoning complexity condition. The correct answers are square, cross and cross, respectively, for the binary, ternary and quaternary problems illustrated. On null trials no reasoning was required. B. Example trial sequence. Each trial contained a jittered fixation period, followed by an LST item, a second, jittered fixation period, a response screen, and a confidence rating scale. In null trials the motor response screen had one geometric shape replaced with an asterisk, which indicated the button to press for that trial. C. LST performance accuracy as a function of reasoning complexity for the two groups (controls on the left and CCD group on the right). Values for each individual are plotted for the controls (gray) and CCD individuals (colors). Black horizontal line-segments represent the mean value for each group and condition. * indicates p < 0.05, ** indicates p < 0.01; Wilcoxon rank sum test.

The LST was identical to the task used in our previous work (Hearne et al., 2017). Each trial involved the presentation of a 4 × 4 matrix populated with a small number of geometric shapes (square, circle, triangle or cross), blank spaces and a single target question mark (‘?’, see Fig. 2A). Participants were asked to solve for the cell containing the question mark, adhering to the rule: “*each shape can only occur once in every row and once in every column”.* Binary problems require integration of information across a single row *or* column. Ternary problems involve integration across a single row *and* column. Quaternary problems require integration of information across several rows and columns. Null trials involved presentation of an LST grid, but instead of a target probe (‘?’) an asterisk was presented (‘*’) indicating to the participant that no reasoning was required. For the brain imaging analyses, the Null trials were utilized as ‘baseline’ comparison periods (see analysis methods below). Puzzles were presented for five seconds, followed by a two second motor response period and confidence rating (Fig. 2B). In total, 144 LST items were presented in the MRI session across 16 blocks, with 36 items in each condition. Trials were pseudo-randomized such that no two items of the same level of complexity occurred sequentially, and each block had two problems from each level of complexity. Participants were not cued to the complexity of any given trial. Both groups were provided with the same task instructions, and prior to the MRI session all participants completed 20 practice trials of the LST (12 with corrective feedback).

#### 2.2.2 Neuroimaging data acquisition

Imaging data for all participants were acquired using a 7 Tesla Siemens Magnetom whole body MRI scanner fitted with a 32-channel head coil. Structural (T1), functional (fMRI), and diffusion-weighted (DWI) MRI was conducted for each participant.

Details regarding the fMRI protocol have been reported elsewhere (Hearne et al., 2017). Briefly, whole brain T2*-weighted gradient echo images were acquired using an optimized multi-band sequence (acceleration factor of five). In each task run, 1250 volumes were collected with the following parameters: voxel size = 2 × 2 × 2 mm, TR = 586 ms, TE = 23 ms, flip angle = 40°, FOV 121 × 208 × 208 mm, 55 slices.

The structural MRI protocol used an MP2RAGE sequence (voxel size = 0.75 × 0.75 × 0.75 mm, MTX 256 × 300 × 320, TR = 4300 ms, TE = 3.44 ms, TI = 840 / 2370 ms, flip angle 5°, FOV 192 × 225 × 240 mm). The DWI protocol used a stimulated-echo diffusion-weighted echo planar imaging (EPI) sequence (voxel size = 1.55 × 1.55 × 1.55, MTX 142 × 142 × 70, FOV 220 × 220 × 126 mm (0.3 mm axial slice gap), Δ / δ = 110 / 30 ms, TR = 12,000 ms, TE = 54.4 ms). Diffusion space was evenly sampled over two half-shells (32 sample points with *b* = 700 s mm^−2^ and 64 sample points with *b* = 2000 s mm^−2^, respectively). Diffusion gradient directions were generated using an electrostatic energy minimization method. Four *B*_0_ (i.e., *b* ≈ 0 s mm^−2^) images were also acquired (one of which was reverse phase-encoded to correct EPI associated image distortions). DWI data for CCD1 were not available due to a technical problem with the acquisition sequence.

#### 2.2.3 Pre-processing

Functional imaging data were pre-processed using the MATLAB (Mathworks, USA) toolbox Data Processing Assistant for Resting-State fMRI (DPARSF V 3.0, Chao-Gan and Yu-Feng, 2010). DICOM images were first converted to Nifti format and realigned. T1 images were re-oriented, skull-stripped and co-registered to the Nifti functional images. Segmentation (gray matter, white matter and cerebrospinal fluid) and the DARTEL algorithm (Ashburner, 2007) were used to improve the estimation of non-neural signal in subject space. From each gray matter voxel, the following signals were regressed: undesired linear trends, signals from the six head motion parameters (three translation, three rotation), white matter and cerebrospinal fluid (estimated from individual-level seed regions within the CSF and white matter). Single-subject functional images were then normalized and smoothed using DARTEL (4 mm^3^). Data processing steps also involved filtering (0.01–0.15 Hz) at a low frequency component of the blood oxygenation level dependent (BOLD) signal known to be sensitive to both resting state and task-based neural activity (Bassett et al., 2015; Braun et al., 2012; Sun et al., 2004).

The DWI data were processed using MRtrix3 (www.mrtrix.org). The diffusion-weighted images were corrected for EPI distortion, motion, and bias field inhomogeneity before the structural images were registered to the diffusion-weighted images. The co-registered structural images were then decomposed into five tissue type (5TT) images: Cortical gray matter, sub-cortical gray matter, white matter, CSF, and pathological tissue. The 5TT images were used in conjunction with the DWI data to create separate fiber orientation distribution function (fODF) maps for WM, gray matter (GM) and cerebrospinal fluid (CSF) via the multi-shell, multi-tissue, constrained spherical devolution (MSMT-CSD) algorithm, with a maximum spherical harmonic order of eight (Dhollander et al., 2016; Jeurissen et al., 2014).

#### 2.2.4 Task-evoked activity

The mean performance of CCD individuals in the Quaternary condition (Fig. 2C) was at chance level. We therefore excluded data from this condition for the imaging analyses. Regional activity changes associated with increments in relational complexity were isolated using the general linear model (GLM) framework implemented in SPM12 (Friston et al., 1994). Fixation, reasoning (Null, Binary, Ternary), motor response, and confidence rating epochs were each modelled as boxcar functions convolved with a canonical hemodynamic response function. Temporal autocorrelations were corrected using the FAST algorithm due to the short TR (Bollmann et al., 2018).

Single subject (first-level) *t*-contrasts were used to isolate the average positive effect of increased relational complexity [Null (−2) versus Binary (1) and Ternary (1)]. In the control group, first level contrasts were used to undertake a one-sample *t*-test to isolate the average positive task effect at group-level. An initial uncorrected threshold of p < 0.0001 was applied to define clusters of brain regions. Cluster level correction was subsequently ascribed to declare significance [p < 0.001, family-wise error corrected (FWE)]. Considering the small sample size of the CCD cohort, we generated binarized activity maps containing the highest *t*-statistics for each individual (top 5% of values at voxel level). These maps were then overlaid to create a group-level ‘consistency map’. The rationale behind these analyses was to isolate brain clusters involved in LST performance in both groups. Results from the two groups were qualitatively compared (Results section). The SPM toolbox MarsBar (Brett et al., 2002) was subsequently used to extract single-subject beta-values from twelve regions of interest (ROI, 5 mm spheres; Table 2). The ROI were defined based on fMRI activations (see Results) and were given brain network affiliations in line with previous literature (Cocchi et al., 2014; Cole and Schneider, 2007; Dosenbach et al., 2006; Duncan, 2010). Six ROI mapped onto the fronto-parietal network (FPN) and six onto the cingulo-opercular network (CON). The placement of each individual ROI was manually checked to confirm that differences in neuroanatomy within the CCD cohort did not lead to sampling error. To test for changes in neural activity between groups and reasoning complexity, mean beta values for each network were entered into a two (group) by two [complexity (Null versus Binary, Null versus Ternary)] mixed ANOVAs.

**Table 2:** Regions of interest derived from the GLM analysis.

Anatomy	Network	MNI Coordinate			Overlap
		x	y	z	
Right superior frontal	FPN	26	−4	59	6
Left superior frontal	FPN	−26	−3	64	5
Right rostrolateral prefrontal cortex	CON	38	34	25	6
Left rostrolateral prefrontal cortex	CON	−43	31	25	4
Right inferior frontal	FPN	49	8	29	5
Left inferior frontal	FPN	−48	4	29	6
Right anterior cingulate cortex	CON	9	4	45	6
Left anterior cingulate cortex	CON	−6	4	45	4
Right inferior parietal	FPN	54	−32	47	4
Left inferior parietal	FPN	−48	−32	41	6
Right anterior insula	CON	39	23	−7	3
Left anterior insula	CON	−37	24	−7	4

Note: Overlap represents the number of CCD individuals (out of 7) who demonstrated high *t*-statistics (top 5%) at a given MNI coordinate.

#### 2.2.5 Task-evoked functional connectivity

Task-evoked functional connectivity was estimated using the same method adopted in Hearne et al. (Hearne et al., 2017). In short, regionally averaged time-series were extracted for the twelve ROIs, and a task nuisance covariate was regressed from each signal (Cole et al., 2014). After accounting for the hemodynamic lag, the residual values were concatenated to form a condition-specific (Null, Binary, Ternary) time-series of interest. A Pearson correlation was performed on the residual time-series. Connectivity matrices were fisher-Z transformed to improve the use of parametric statistics. To test for differences in functional connectivity across complexity conditions and groups we conducted two (group) by three (complexity) mixed ANOVAs on mean connectivity values in each network on both inter- and intra-hemispheric connections.

#### 2.2.6 Structural connectivity

Structural connectivity analyses were undertaken using MRtrix3 (Tournier et al., 2012). First, CSD-based probabilistic tractography (using the iFOD2 algorithm) (Tournier et al., 2010) was used to generate whole-brain tractograms for each participant. The tractograms consisted of 20 million streamlines each that were seeded randomly across the whole-brain white matter. The streamlines were anatomically constrained to the WM to ensure streamlines did not path through GM, CSF, or potential lesions (Smith et al., 2012). The tracking parameters were: fODF cut-off = 0.1, step size = 0.5 mm, curvature threshold = 45°/step, minimum / maximum streamline length = 10 / 200 mm. These tractograms were then parsed by a CSD-informed tractogram filter (Smith et al., 2013) to create a new tractogram consisting of the 2 million streamlines that represented the most likely propagations of fiber tracts. Binary connectomes (matrix representations of the brain network topology) were generated from the filtered tractograms using the Harvard-Oxford cortical and subcortical structural parcellation atlas (Desikan et al., 2006; Frazier et al., 2005; Goldstein et al., 2007; Makris et al., 2006). The atlas was warped into the native space for each set of diffusion-weighted images by co-registering the associated MNI 152 template to these images. To estimate the extent of inter- and intra-hemispheric structural connectivity, the number of connections (i) within and (ii) across hemispheres was summed across ipsilateral and contralateral paired regions in each connectome (Fig. 1B)*.* Further analysis taking connection weight into account showed similar results.

## 3 Results

### 3.1 Behavior

For the LST, a typical complexity effect was observed in individuals with CCD, as previously found in independent samples of neurotypical adults (Birney and Bowman, 2009; Cocchi et al., 2014) (Fig. 2C). Reasoning problems of greater complexity resulted in significantly more reasoning errors (*F*_2,12_ = 60.45, p < 0.001, *n*^2^_*p*_ = 0.91) and longer reaction times (RT; *F*_2,12_ = 6.31, p = 0.013, *n*^2^_*p*_ = 0.51). Follow-up tests confirmed that participants were generally more accurate, and faster, in the Binary than in the Ternary condition (accuracy: *t*_6_ = 4.15, p = 0.006, *d* = 1.57, RT: *t*_6_ = 3.89, p = 0.008, *d* = 1.47), and in the Ternary than in the Quaternary condition (accuracy: *t*_6_ = 6.89, p < 0.001, *d* = 2.60; RT: n.s., *t*_6_ = 0.67, p = 0.53, *d* = 0.25) (Fig. 2C).

When compared with controls, a mixed ANOVA (complexity × group) revealed a significant main effect of group for accuracy (*F*_2,70_ = 27.26, p < 0.001, *n*^2^_*p*_ = 0.44) and RT (*F*_2,70_ = 9.27, p = 0.004, *n*^2^_*p*_ = 0.21), such that controls were faster and more accurate than CCD individuals. The CCD cohort showed reduced accuracy and slower reaction times compared to controls at all complexity levels (p < 0.05). No interaction was observed for either accuracy or RT (Fig. 2C). CCD individuals also showed reduced performance on the APM (M = 7.86, SD = 2.34) compared with controls (M = 10.17, SD = 1.37, *z* = 2.47, p = 0.01), which is consistent with previous work suggesting a relationship between the LST and fluid intelligence (Birney and Bowman, 2009; Hearne et al., 2017). Overall, the behavioral results suggest that CCD individuals were slower and more error-prone than controls but showed an analogous complexity-induced decline in accuracy and increase in response speed. Four CCD individuals performed at chance level in the Quaternary trials (Fig. 2C), and so this condition was eliminated from the imaging analyses.

### 3.2 Brain activity

**Fig. 3:**
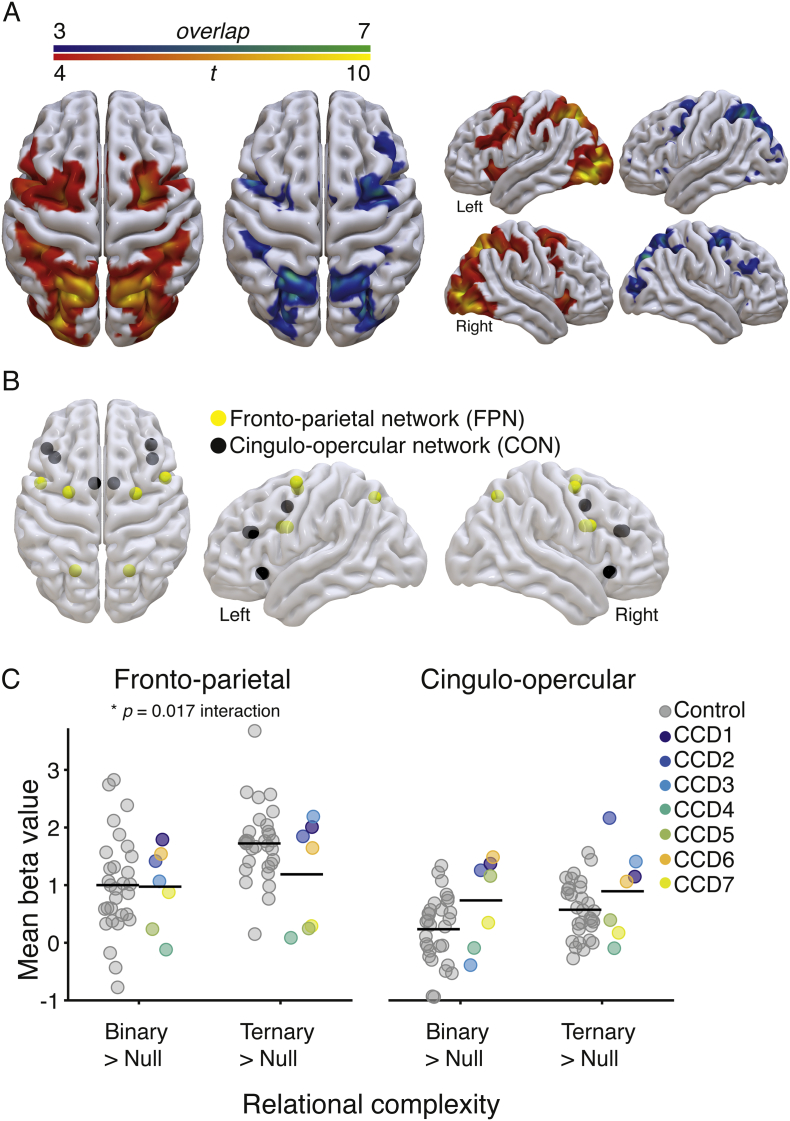
Brain activity associated with increases in reasoning complexity. A. Brain activity evoked in the CCD group (*N* = 7), comparing null versus binary and ternary conditions, contrasted with brain activity evoked in the control group (*N* = 30). The blue-green heat map (left) represents the overlap of brain activity across CCD individuals (voxel level top 5% of *t*-values), such that green represents complete overlap (100%, *N* = 7) and blue represents less overlap (42%, *N* = 3). The red-yellow heat map (right) shows the results of a random effects analysis in the control group (conservative threshold of p < 0.0001 uncorrected, p < 0.001 FWE corrected at cluster level). This stringent statistical threshold was adopted to highlight the key neural clusters involved in the task. Brain renderings were created using SurfIce software (www.nitrc.org/projects/surfice). B. Regions of interest (ROIs) plotted as spheres in a glass brain (details in Table 2). These brain regions overlap with the complexity-induced patterns of activity shown by the CCD and control participants, and comprise two well-known brain networks involved in cognitive control: the fronto-parietal (FPN) and cingulo-opercular (CON) networks (Cocchi et al., 2014; Dosenbach et al., 2006; Duncan, 2010; Seeley et al., 2007; Yeo et al., 2011). C. Change in the estimated beta values averaged across ROIs within the FPN (left) and CON (right). Values for each individual are plotted for the control (gray) and CCD cohort (color, legend in center). Black horizontal line-segments represent the mean value for each group and condition.

We first tested whether individuals with CCD showed similar brain activity patterns as the control group in response to reasoning complexity. Previous work on cognitive reasoning has isolated co-activations within fronto-parietal (FPN) and cingulo-opercular networks (CON) that respond to task complexity, such that increased complexity is coupled with an increase in the BOLD response (Cocchi et al., 2014; Cole and Schneider, 2007; Dosenbach et al., 2006; Duncan, 2010). We calculated the average positive effect of increased relational complexity by comparing Null versus Binary and Ternary conditions in a general linear model (see Methods for details). Due to the small sample size of the CCD cohort, we generated binarized activity maps containing the highest *t*-statistics for each individual (voxel level, top 5% of values; range 0.7–27.5). These maps were then overlaid to create a ‘consistency map’ (shown in Fig. 3A). Qualitatively, the resulting maps suggest that both groups share a similar pattern of neural co-activations in response to reasoning task demands, involving key cortical clusters in regions of the FPN and CON. Mean and standard deviation SPM t-statistic maps for each group and condition are shown in Fig. S1.

To investigate the magnitude of brain activation across conditions, we compared change in neural activity (beta values) within twelve regions of interest that were selected based upon the identified patterns of activity (Fig. 3B & Table 2). A mixed ANOVA (complexity × group) on mean change in beta values across each brain network revealed a significant main effect of complexity, such that the Ternary condition yielded a larger increase in neural activity than the Binary condition when compared with the Null condition (FPN: *F*_1,35_ = 21.45, p < 0.001, *n*^2^_*p*_ = 0.38; CON: *F*_1,35_ = 6.2, p = 0.02, *n*^2^_*p*_ = 0.15; Fig. 3C). For the FPN, a significant complexity by group interaction was observed (*F*_1,35_ = 6.30, p = 0.017, *n*^2^_*p*_ = 0.15). This interaction suggests that, contrary to controls, individuals with CCD showed a diminished increase in neural activity from Binary to Ternary conditions within the FPN (Fig. 3C).

### 3.3 Brain connectivity

Having characterized changes in complexity-induced brain activity, we next tested whether individuals with CCD demonstrated similar patterns of inter- and intra-hemispheric task-based functional connectivity to the control cohort in response to increasing relational complexity. Previous work has shown that increased relational complexity induces robust increases in functional connectivity between the FPN and CON (Cocchi et al., 2014). Task-based functional connectivity was calculated between regions that showed the most consistent BOLD activation across all participants (Table 2).

**Fig. 4:**
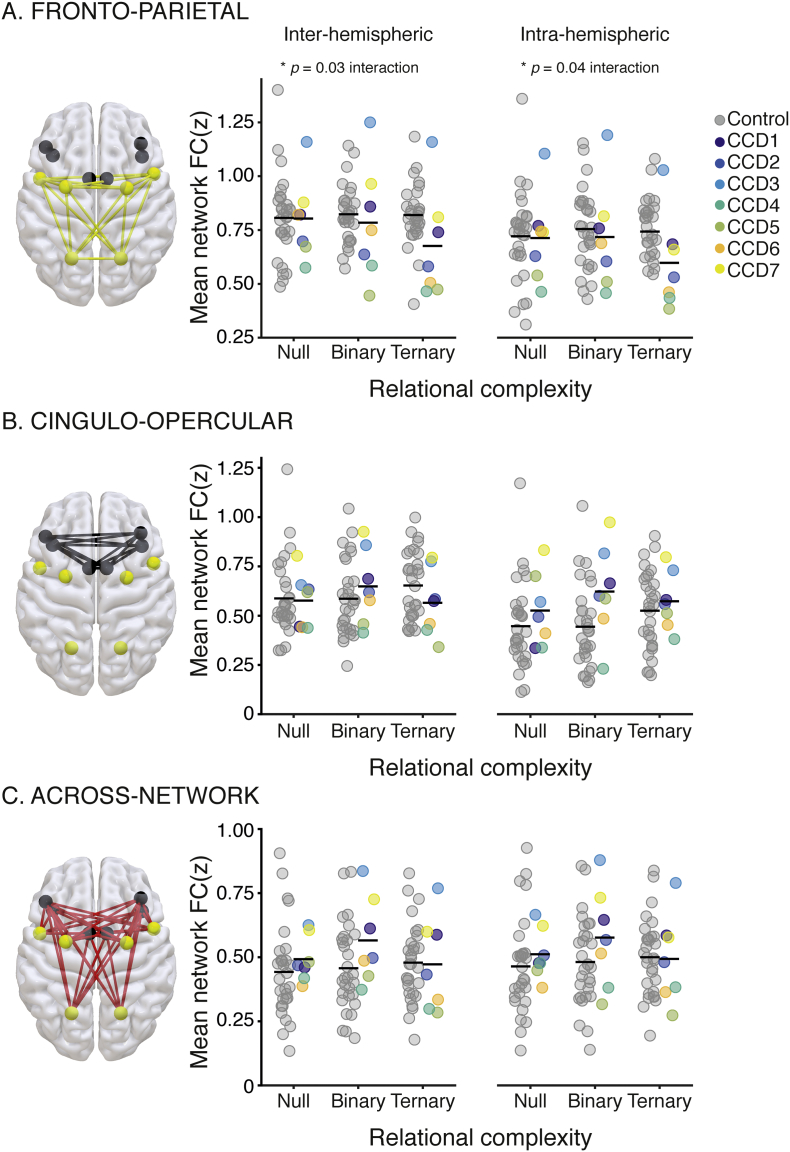
Within- and across-network functional connectivity associated with increasing reasoning complexity. Mean inter-hemispheric (left) and intra-hemispheric (right) *z*-transformed functional connectivity values (*y-axis*) plotted as a function of increasing reasoning complexity demands (*x-axis*: Null, Binary, Ternary). A. Fronto-Parietal (FPN) connections. B. Cingulo-Opercular (CON) connections. C. Across-network connections. Brain renderings of the included connections are on the right (yellow for FPN, black for CON, and red for across-network connections). Values for each individual are plotted for the controls (gray circles) and CCD individuals (colored dots, legend top middle). Black horizontal line-segments represent the mean value for each group and condition.

The CCD cohort had similar levels of mean functional connectivity to controls in the Null condition, supporting previous work showing intact bilateral networks in CCD individuals under low cognitive load (Owen et al., 2013; Tyszka et al., 2011). By contrast, a mixed ANOVA with factors of complexity and group revealed an interaction in the FPN (inter-hemispheric: *F*_2,70_ = 3.69, p = 0.030, *n*^2^_*p*_ = 0.10, intra-hemispheric: *F*_2,70_ = 3.37, p = 0.040, *n*^2^_*p*_ = 0.09, Fig. 4A). In this network, CCD individuals showed a reduction in functional connectivity as a function of complexity compared with controls (Fig. 4A).

### 3.4 Control analysis

**Fig. 5:**
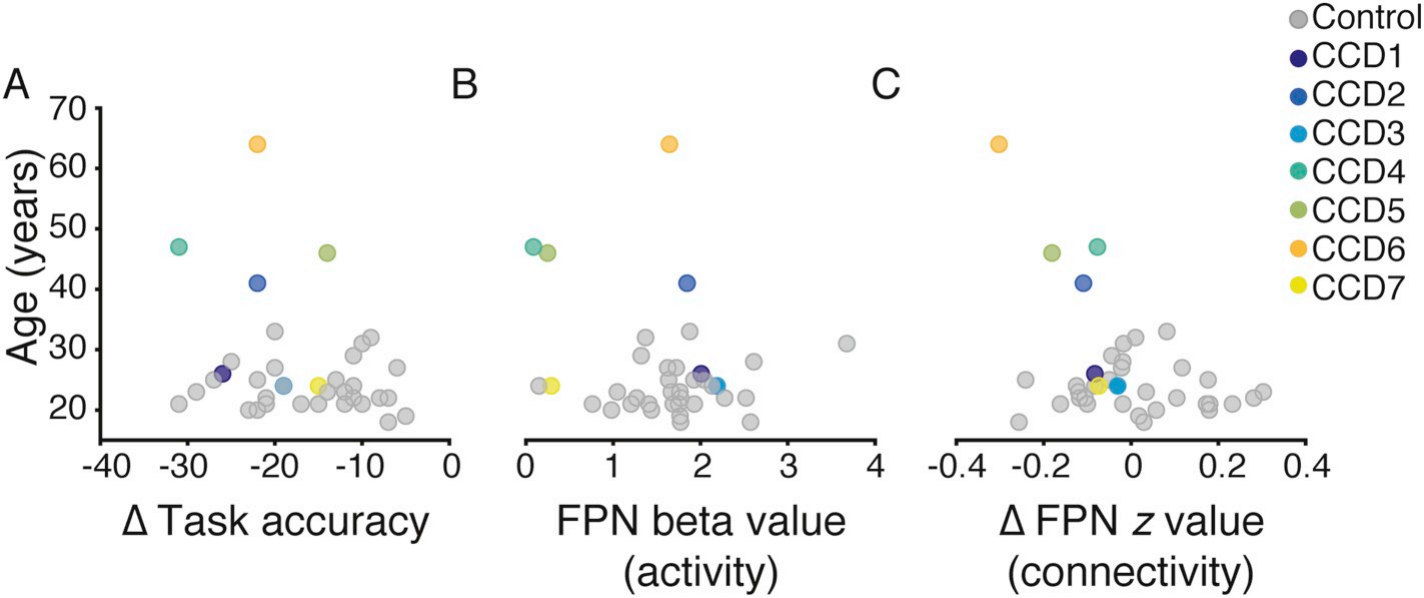
Control analyses to investigate the effect of age. Scatter plots of changes in task accuracy (A), FPN activity (B), and FPN connectivity (C) plotted as a function of participant age (*y*-axis).

A Wilcoxon rank sum test revealed differences in age between individuals with CCD and the control cohort (M_CCD_ = 38.86, SD_CCD_ = 15.06, M_HC_ = 22.29, SD_HC_ = 5.25, *z* = −3.36, p < 0.001). As a control analysis we plotted changes in task accuracy (Ternary – Binary) beta values (Ternary – Null) and functional connectivity (Ternary – Null) as a function of individuals' age. Numerically, values from the oldest CCD participants were within the range of younger healthy controls. For example, in Fig. 5C the oldest CCD participant shows the lowest connectivity value (z-value), while the next lowest value is from an 18-year-old healthy control. These analyses suggest that between group differences in age are unlikely to explain our findings.

## 4 Discussion

Despite the absence or malformation of the corpus callosum, preserved bilateral functional brain networks have been observed in individuals with CCD (Owen et al., 2013; Tovar-Moll et al., 2014; Tyszka et al., 2011). These whole-brain functional networks are thought to be supported by atypical interhemispheric white matter pathways that form during development (Lassonde et al., 1991; Tovar-Moll et al., 2014). Less is known about the role and functional properties of brain networks supported by this abnormal connectome. Here we had individuals with CCD undergo fMRI while solving non-verbal reasoning problems known to elicit robust neural responses within and between the FPN and CON (Cocchi et al., 2014; Crittenden et al., 2016; Hearne et al., 2015; Hearne et al., 2017). Behaviorally, individuals with CCD showed a typical complexity effect, in that their performance declined as a function of increased reasoning complexity (Birney and Bowman, 2009; Hearne et al., 2017). Neural activity and connectivity patterns within and between the FPN and CON networks in CCD individuals were largely preserved in low-complexity trials. By contrast, abnormal patterns of activity and connectivity emerged in this group under higher complexity conditions, specifically within the FPN. These findings reveal previously unknown state-dependent deficits of functional integration in individuals with CCD, which evidently remain hidden at rest or when cognitive demands are low.

Our results support the notion that resting-state functional networks underpinning higher cognitive processes can be supported by abnormal configurations of the connectome (Mišić et al., 2016; Owen et al., 2013; Tyszka et al., 2011). Under low complexity conditions (i.e., the Null or Binary problems of the LST), we observed relatively normal neural activity and patterns of functional connectivity within and between segregated brain regions of the FPN and CON in CCD individuals (Fig. 3, Fig. 4). Strikingly, even participants with complete agenesis of the corpus callosum showed bilateral patterns of activity and connectivity in these networks. Subcortical structures, as well as the anterior and posterior commissures (Tovar-Moll et al., 2014), can provide common input to both cerebral hemispheres, leading to observable bilateral functional neural networks. The thalamus, for example, is endowed with the type of whole-brain structural and functional connectivity necessary for such a role (Behrens et al., 2003; Bell and Shine, 2016; Hwang et al., 2017; Schmitt et al., 2017), as well as the potential to support direct inter-hemispheric communication through the interthalamic adhesion (Damle et al., 2017). Our findings in CCD individuals provide motivation for further work to assess the putative contribution of subcortical routes in supporting bilateral brain activity in pathologies that impact the corpus callosum.

Under conditions of high reasoning demands, however, we found that individuals with CCD showed decreased local neural activity and functional connectivity within the FPN. In terms of brain activity, individuals with CCD showed a smaller increase in neural activity compared to controls when attempting to solve more complex reasoning problems. Moreover, CCD individuals showed a decline in functional connectivity within the FPN as reasoning complexity increased, whereas controls instead showed a shift toward increased functional connectivity with increasing cognitive load across both the FPN and CON (Fig. 4) (Cocchi et al., 2014). Critically, we found little distinction between intra- and inter-hemispheric connectivity in regards to this deficit, suggesting a global impact of CCD on task-induced FPN dynamics. The FPN is thought to be responsible for the moment-to-moment implementation of cognitive task goals (Cocchi et al., 2013; Dosenbach et al., 2007). Damage to frontal and parietal cortices has also been associated with poorer performance in tests of cognitive reasoning (Urbanski et al., 2016; Woolgar et al., 2010). In line with this, our results suggest that in CCD individuals FPN activity does not modulate in the manner required to solve complex cognitive problems.

CCD is a developmental malformation of the central nervous system, affecting approximately 1:4000 people (Glass et al., 2008). The current study included seven individuals with CCD recruited from Australia over a period of 18 months. While this sample size is comparable to, or larger than, that of several previous investigations (Owen et al., 2013; Quigley et al., 2003; Tovar-Moll et al., 2007, Tovar-Moll et al., 2014; Tyszka et al., 2011; Wahl et al., 2009), it was nevertheless too small for us to use many of the conventional analytic approaches for inferring differences in neural activity and connectivity between groups (Eklund et al., 2016; Friston et al., 1994; Zalesky et al., 2010). We therefore opted for an analysis strategy that balanced statistical rigor with the reality of describing whole-brain neural networks in a rare and heterogeneous condition. Group by complexity interactions for connectivity values did not quite survive a strict correction for multiple comparisons. However, altered complexity-induced changes in connectivity were consistent with the observed abnormalities in FPN activity. Our control analyses argue against a major impact of age or handedness (see Fig. S2) on the results, but we cannot rule out a possible influence of other potential confounding variables on the observed interaction. Likewise, CCD has been associated with additional heterogeneous cerebral malformations including, for example, abnormal sulcation, noncallosal midline abnormalities, and differences in axonal demyelination, that likely influence behavioral and functional imaging outcomes (Barkovich and Norman, 1988; Hetts et al., 2006; Parrish et al., 1979). Finally, due to the small sample size the possibility of more subtle and undetected deficits in complexity-induced network activity in CCD cannot be excluded. It is important to consider the current findings as initial evidence to guide further, more definitive research with larger sample sizes.

In conclusion, the current findings are in line with the notion that resting-state functional brain networks supporting cognitive control are at least partially preserved in individuals with CCD (Tyszka et al., 2011). This observation also provides an empirical demonstration of the brain's ability to accommodate canonical functional networks within different anatomical scaffolds (Mišić et al., 2016). Despite preserved network activity and functional connectivity under low-complexity reasoning demands, the FPN in individuals with CCD did not seem to effectively accommodate increasingly complex task demands. This suggests that, even with alternative pathways to support brain activity, deficits in corpus callosum integrity impose a hard limit on the capacity of the FPN to dynamically adapt its activity to high cognitive demands. When considered in conjunction with individual differences in structural connectivity, task-driven deficits in FPN integration provide a plausible neural mechanism to explain the broad and heterogeneous cognitive deficits commonly observed in individuals with CCD.

## Conflicts of interest

None.

## Appendix A Supplementary data

Supplementary material Image 1
