# Supplementary material for: Increased cognitive complexity reveals abnormal brain network activity in individuals with corpus callosum dysgenesis
Source: Neuroimage Clin. 2018 Nov 14;21:101595. doi: 10.1016/j.nicl.2018.11.005 (PMC6411589; doi:10.1016/j.nicl.2018.11.005)
Supplement: Supplementary file 1 — Supplementary material [file mmc1.docx]

# Supplementary Information

# Increased cognitive complexity reveals abnormal brain network activity in individuals with corpus callosum dysgenesis

Luke J. Hearne^a^, Ryan J. Dean^a^, Gail A. Robinson^bd^, Linda J. Richards^a,c^, Jason B. Mattingley^a,b*^, Luca Cocchi^d*^

^a^ Queensland Brain Institute, The University of Queensland, Brisbane, Queensland, Australia.

^b^ School of Psychology, The University of Queensland, Brisbane, Australia.

^c^ School of Biomedical Sciences, The University of Queensland, Brisbane, Australia.

^d^Clincal Brain Network Group, QIMR Berghofer Medical Research Institute, Brisbane, Queensland, Australia.

** Shared last author*

Corresponding author:

Luca Cocchi, QIMR Berghofer Medical Research, 300 Herston Road, Brisbane, QLD, 4006. Phone: +61738453008, Email: [Luca.Cocchi@qimrberghofer.edu.au](mailto:Luca.Cocchi@qimrberghofer.edu.au)

**
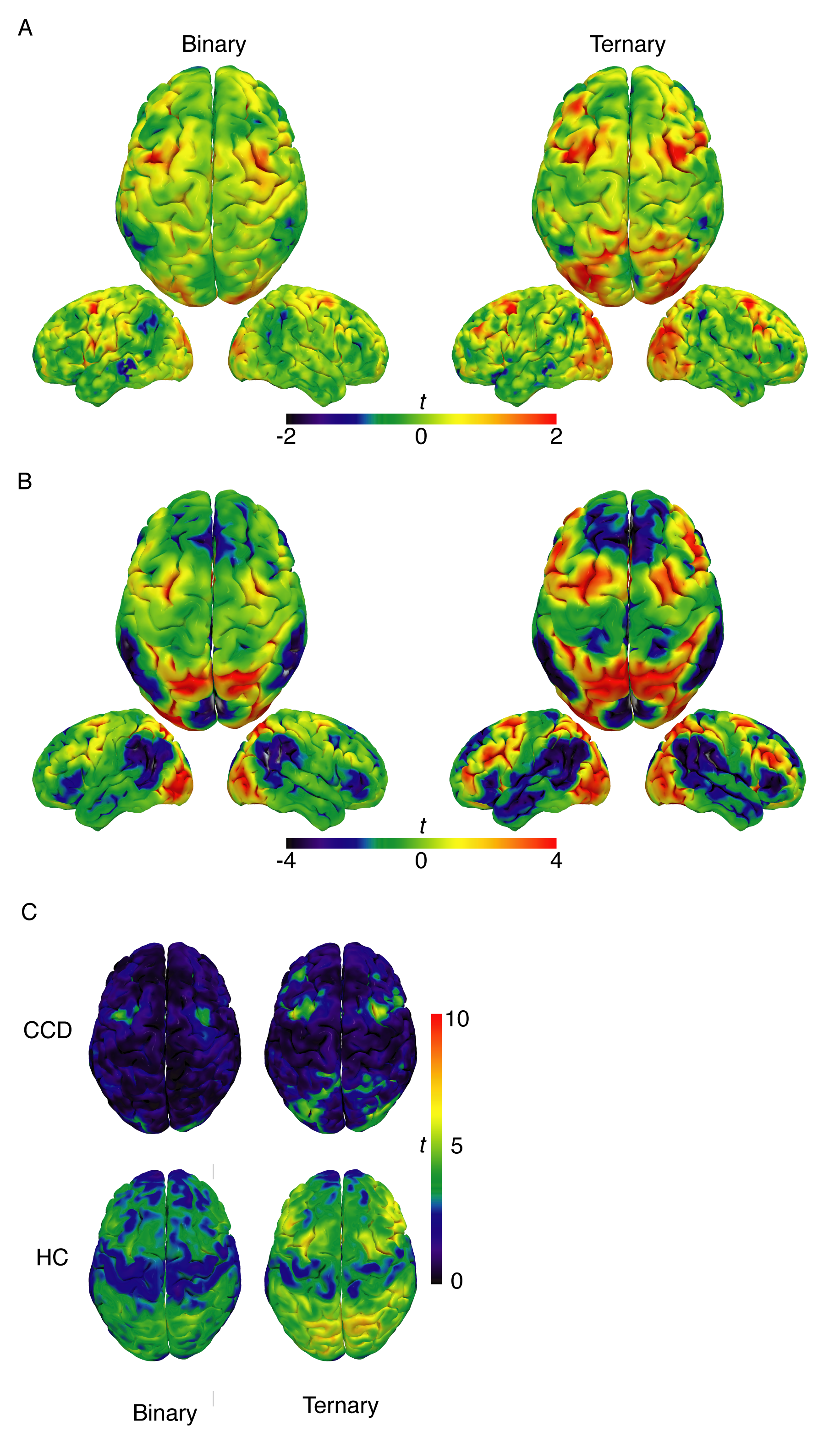
Figure S1.** *Mean and standard deviation t-statistic activation maps*. **A.** Mean activation maps for Binary (left) and Ternary (right) conditions versus Null for the corpus callosum dysgenesis (CCD) group. **B.** The identical contrast in the healthy control (HC) group. These maps were created by averaging the SPM *t*-maps at every voxel across participants. **C.** The standard deviation for each voxel.

**
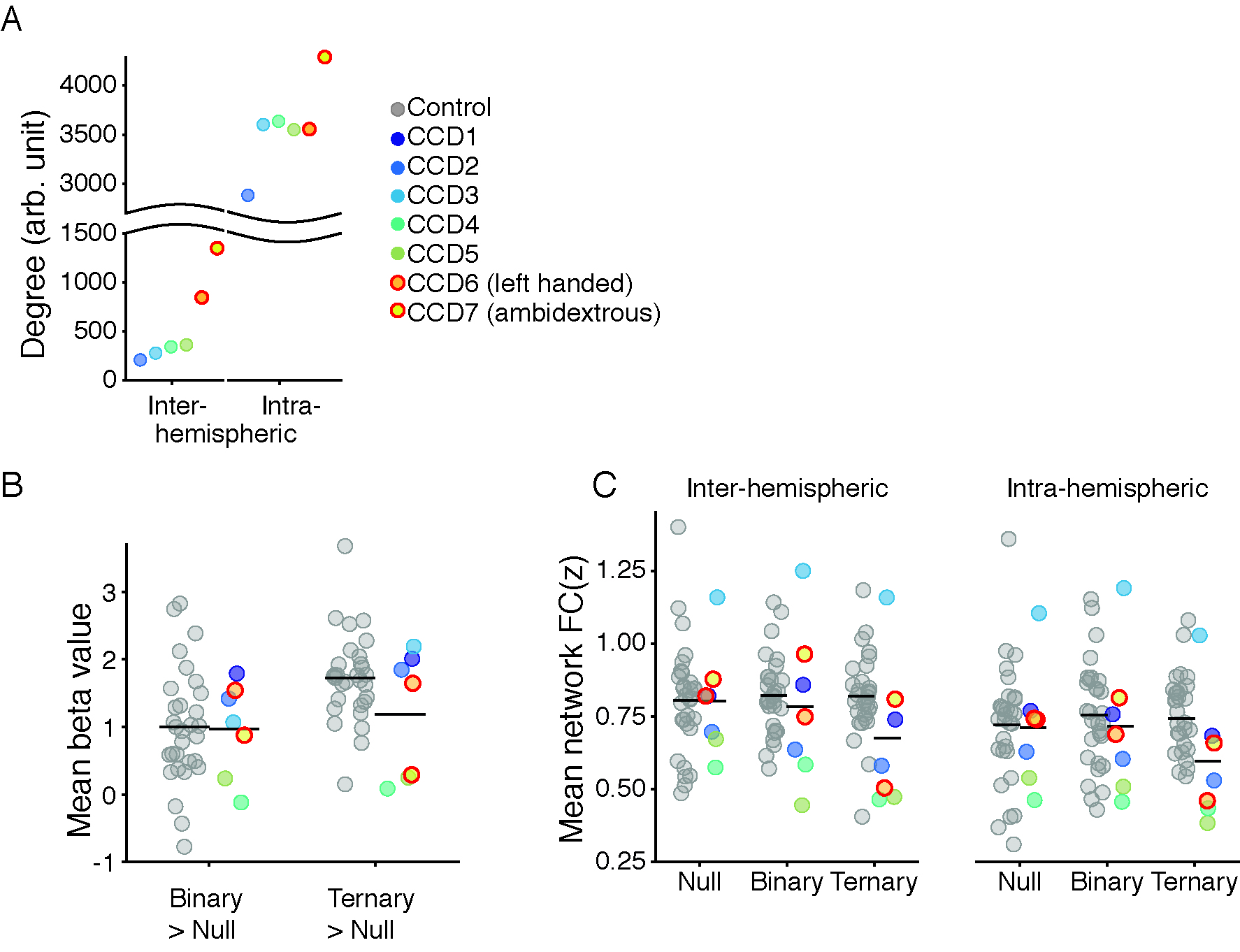
**

**Figure S2.** *Current brain imaging results with handedness highlighted*. **A.** Inter- and intra-hemispheric connectivity indices with the left handed (CCD6) and ambidextrous participants (CCD7) highlighted. **B.** and **C.** show the activity and connectivity results (FPN) from the paper with additional highlights. Neither highlighted subjects represent outlier data in the functional imaging (main results) of the paper.
